# Circulating hsa_circ_0072309, acting via the miR‐100/ACKR3 pathway, maybe a potential biomarker for the diagnosis, prognosis, and treatment of brain metastasis from non‐small‐cell lung cancer

**DOI:** 10.1002/cam4.6371

**Published:** 2023-07-26

**Authors:** Xiao‐Qiang Zhang, Qian Song, Lin‐Xiang Zeng

**Affiliations:** ^1^ Department of thoracic surgery The Second Affiliated Hospital of Nanchang University Nanchang China; ^2^ Department of Respiratory and Critical Care Medicine The Second Affiliated Hospital of Nanchang University Nanchang China

**Keywords:** diagnostic biomarker, miR‐100/ACKR3 signal, NSCLC‐associated brain metastasis, serum hsa_circ_0072309, therapeutic target

## Abstract

**Background:**

One of the main causes of lung cancer‐related death is brain metastasis (BM). Finding early indicators of BM derived from lung cancer is crucial. Therefore, this study was designed to determine if serum hsa_circ_0072309 may be employed as a potential biomarker for BM induced by non‐small‐cell lung cancer (NSCLC) and to understand its possible underlying mechanism.

**Methods:**

Primary lung cancer and healthy neighboring tissues were obtained from all patients, while BM tissues were taken from BM+ patients. Serum specimens were collected from all patients and healthy volunteers. Hsa_circ_001653, miR‐100, and ACKR3 RNA expressions were analyzed by quantitative reverse transcription‐polymerase chain reaction **(**qRT‐PCR**)**, and atypical chemokine receptor 3 **(**ACKR3**)** protein expression by western blotting (WB), immunohistochemistry (IHC), and enzyme‐linked immunosorbent assay (ELISA). In order to examine the effect of serum hsa_circ_0072309 and its relevant mechanism on BM development, an NSCLC‐associated BM model in mice was established.

**Results:**

According to the results, miR‐100 expression was down‐regulated in primary lung cancer tissues compared to healthy lung tissues in all NSCLC patients, and circ_0072309 and ACKR3 expression were up‐regulated. In BM tissues compared with primary lung tumors of BM+ patients, in serum samples from all patients compared to healthy volunteers, and in lung tumors of BM+ patients compared to those from BM− patients. Patients' serum exhibits the same level of hsa_circ_0072309/miR‐100/ACKR3 expression as in BM samples. Advanced tumor‐node‐metastasis (TNM) stage, higher BM, shorter post‐operative overall survival (OS), and progression‐free survival (PFS) are all substantially associated with increased serum circ_0072309 levels in BM+ patients. In animal models, serum owning hsa_circ_0072309 from BM+ patients facilitates BM formation by regulating the miR‐100/ACKR3 pathway.

**Conclusions:**

The current preliminary research reveals serum hsa_circ_0072309 as a possible biomarker and target for early diagnosis, prognosis, and therapy of NSCLC‐derived BM and suggests a substantial role for the hsa_circ_0072309/miR‐100/ACKR3 axis in the formation of BM from NSCLC.

## 1 INTRODUCTION

Lung cancer (LC) is the most common malignant tumor of the respiratory system, and non‐small cell lung cancer (NSCLC) accounts for about 85%–90%.^1^ The most common metastatic tumor in the brain is brain metastasis (BM), which develops from LC, primarily from NSCLC, and has an incidence rate of 23% to 65%.^2^, ^3^ About 19% of NSCLC patients experienced concurrent BM at the time of their first diagnosis, and most exhibited multiple metastases.^4^ Studies over the past 20 years showed that the 5‐year survival rate of patients with advanced NSCLC is less than 10%, and about 50% of patients have BM.^5^ LC‐derived BM is highly invasive and has a very poor prognosis.^6^ Most patients do not survive more than 3–6 months after the initial diagnosis due to BM, which is the primary reason for LC treatment failure and death.^7^, ^8^ Thus, early detection and treatment of BM are critical to alleviating patient pain and prolonging survival. Further studies on tumor biomarkers suggest that they may have specific values for early diagnosis and prognosis of LC.^9^ Additionally, emerging data indicated that investigating serological tumor markers for BM from LC is crucial for the early identification and rational treatment decision‐making of LC‐derived BM.^10^, ^11^, ^12^ Among these, circular RNAs (circ RNAs) have been demonstrated to be novel biomarkers for diagnosis, prognosis, and therapy in human cancers because they are difficult for RNA enzymes in bodily fluids to break down. Circular RNAs have consequently emerged as promising clinical practice biomarkers.^13^, ^14^ Therefore, the main objective of this study is to identify a new circ RNA marker from peripheral blood that can be utilized for diagnosis, prognosis assessment, and treatment of BM from NSCLC, as well as to investigate the probable underlying molecular mechanisms.

Atypical Chemokine Receptor 3 (ACKR3) is a seven transmembrane domain receptor belonging to class A G‐protein‐coupled receptors and is overexpressed in numerous cancer types, including LC, and is closely associated with poor prognosis.^15^ According to earlier research, ACKR3 can be a biomarker for post‐operative 5‐year disease‐free survival in stage I NSCLC patients. Higher levels of ACKR3 were found in lung samples from patients who experienced a significantly worse 5‐year progression‐free survival (PFS) rate after surgery.^16^ ACKR3 can also be found in brain and cerebrovascular tissues, where high expression is associated with poor prognosis in brain cancers such as glioma.^15^ In 56 patients with BM, C‐X‐C motif chemokine receptor 7 (CXCR7) was expressed in tumor and endothelial cells within the tumor and in adjacent brain tissues. Due to enhanced adhesion between CXCR7 positive tumor cells and other CXCR7 positive cells in the BM lesions, CXCR7 may make tumors more likely to penetrate the blood–brain barrier.^17^ C‐X‐C motif chemokine ligand 12 (CXCL12), a CXCR7 ligand, is involved in BM induced by several cancers. A study of 32 surgically resected NSCLC patients revealed that CXCL12 immunoreactivity is considerably higher in patients with BM.^18^ These findings suggest that ACKR3/CXCR7 may predict BM in LC.

MicroRNAs are involved in many aspects of tumorigenesis. MiR‐100 can inhibit tumor cell proliferation, growth, and metastasis by regulating ACKR3/CXCR7.^19^ MiR‐100 is downregulated in NSCLC tissues and is negatively associated with clinical stage, tumor classification, lymph node metastasis, and poor prognosis (overall survival, OS). Overexpression of miR‐100 inhibits NSCLC cell development by halting cells in the G2/M phase and increasing apoptosis. As a result, it is believed to be a potential molecular diagnostic and prognostic marker for NSCLC.^20^ MiR‐100 is downregulated in brain cancers such as glioblastoma, and its overexpression plays an inhibitory role.^21^, ^22^ These results suggest that miR‐100 may play an important role in the BM of NSCLC by targeting ACKR3/CXCR7.

The assessments of serological tumor markers for BM resulting from LC are critical in the early detection and sensible treatment selection for this condition.^10^, ^11^, ^12^ Blood biomarker detection offers the advantage of being simple to use and improving patient compliance. Circular RNAs (circRNAs) resist breakdown by RNA enzymes in body fluids. They may be valuable novel biomarkers for diagnosing, prognosis, and treating human cancers, making them perfect for clinical application.^13^, ^14^ Studies have shown that the circRNA, hsa_circ_0072309, is highly expressed in brain diseases such as ischemic stroke, and its low expression can promote apoptosis by targeting miRNA‐100.^23^ Microarray analysis showed that hsa_circ_0072309 is highly expressed in NSCLC tissues and promotes proliferation, migration, and invasion of NSCLC.^24^ As a result, we hypothesize that hsa_circ_0072309 may play a role in NSCLC‐derived BM by targeting the miRNA‐100/ACKR3 axis and that it may be relevant for the diagnosis, prognosis, and therapy of NSCLC‐derived BM. However, it is unknown if hsa_circ_0072309 is expressed in the peripheral blood of NSCLC patients with and without BM. Consequently, we sought to determine if serum hsa_circ_0072309 may be used as a biomarker for diagnosing, prognosis, and treating NSCLC‐derived BM. Moreover, to explore whether its mechanism is related to targeted control of the miRNA‐100/ACKR3 axis.

## 2 MATERIALS AND METHODS

### 2.1 Patients, clinical specimens, and clinical data

120 NSCLC patients (lung adenocarcinoma) were divided into two groups: 60 with BM (BM+) (60 with wild‐type EGFR and mutant KRAS, 32 with wild‐type ALK and 28 with mutant ALK) and 60 without BM (BM−) (60 with wild‐type EGFR and mutant KRAS, 30 with wild‐type ALK and 30 with mutant ALK). 60 healthy volunteers/healthy controls (HC) who participated in physical examinations at the Second Affiliated Hospital of Nanchang University from July 2021 to July 2022 and 60 patients who received complete surgical resection were included in this study. The inclusion criteria were as follows: (1) all patients were confirmed pathologically with NSCLC and identified as BM+ or BM− by computed tomography (CT), positron emission tomography (PET)‐CT, or magnetic resonance imaging (MRI); (2) surgery can achieve complete resection (multiple BM foci must be concentrated in areas of the brain with non‐critical functions and can also be completely resected) with the intent to cure; (3) the eastern cooperative oncology group (ECOG) score is 0–1; (4) no blood diseases; (5) no other malignant tumors; (6) no primary brain cancers. The exclusion criteria were: (1) patients with dual, multiple, or secondary lung cancer; (2) undergoing chemotherapy, radiation, or immunotherapy before surgery.

The specimens collected include tissue and blood. Tissue specimens of BM+ patients include primary lung carcinoma foci (BM+ lung), BM foci (BM+ brain), and normal adjacent lung tissues (normal lung). Tissue specimens of BM− patients include primary lung cancer foci (BM− lung) and normal neighboring lung tissues (normal lung). Peritumor normal tissue is defined as tissues from the surgical section stump that are at least 2 cm distant from the malignant tissues and are certified as healthy by post‐operative pathological analysis. Tissue samples were separated into two parts: paraffin embedding and immunohistochemistry (IHC) and straight freezing in liquid nitrogen and storage until further examination. Post‐fasting peripheral venous blood (5 mL) was collected between 7:30 and 9:30 am from NSCLC patients before tumor resection and 1‐, 2‐, 3‐ and 5‐years post‐operation, using test tubes without anticoagulant. Blood samples from healthy volunteers were collected at a one‐time point only. Serums were extracted by high‐speed centrifugation of the whole blood samples and stored at −80°C until analysis.

Age, gender, tumor stage according to the Union for International Cancer Control (UICC) staging standards and the 7th edition of the tumor‐node‐metastasis (TNM) standard, smoking history, family history of lung cancer, and number of BM foci from CT imaging were obtained from the patient's clinical records. During the post‐operative follow‐up visits, the data on overall survival (OS) and PFS in post‐operative years (1–3, and 5) were recorded.

### 2.2 Quantitative reverse transcription‐polymerase chain reaction (qRT‐PCR)

The RNA expression of hsa_circ_0072309, miR‐100, and ACKR3 in tissue and serum samples were examined through qRT‐PCR, according to the manufacturer's directions. Total RNA was extracted from each sample using Trizol reagent (Invitrogen) and quantified employing a Nanodrop 2000 spectrophotometer (Thermo Fisher Scientific, Inc.). The PrimeScript™ RT reagent Kit (TaKaRa) was used for qPCR on total RNA (1 μg), and reverse transcription kits (Promega Corporation) were used to synthesize cDNA. For miRNA, cDNA was synthesized with the NCode™ VILO™ miRNA cDNA synthesis kit (Invitrogen), and qPCR was performed with the EXPRESS SYBR®‐GreenER™ miRNA RT‐qPCR kit (Invitrogen). All qPCR reactions were executed on the ABI 7500 Real‐Time PCR system (Bio‐Rad), and the 2^−ΔΔCt^ method was used for quantification. β‐actin and U6 were used as endogenous controls for normalization of ACKR3/hsa_circ_0072309 and miR‐100 expression, respectively. Table 1 contains the gene primer sequences used in the current study.

**TABLE 1:** The sequences of the gene primers.

Gene name	Primer sequences	
	Sense	Antisense
hsa_circ_0072309	TCCACACCGCTCAAATGTTA	ATCCAGGATGGTCGTTTCAA
ACKR3	GGCTATGACACGCACTGCTACA	TGGTTGTGCTGCACG AGACT
miR‐100	GAGCCAACCCGTAGATCCGA	GTGCAGGGTCCGAGGT
β‐actin	CTCCATCCTGGCCTCGCTGT	CTCCATCCTGGCCTCGCTGT
U6	CTCGCTTCGGCAGCACA	AACGCTTCACGAATTTGCGT

### 2.3 Western blotting (WB)

Total protein was extracted from tissue samples using RIPA lysis buffer supplied by the Beyotime Institute of Biotechnology in Shanghai, China, and the concentration was calculated using a BCA assay kit from the same company. This procedure evaluated the expression of the ACKR3 protein in the tissue samples. Proteins were separated with 10% SDS‐PAGE (sodium dodecyl sulfate‐polyacrylamide gel electrophoresis), transferred onto polyvinylidene fluoride (PVDF) membranes (Bio‐Rad Laboratories), and immunoblotted for human β‐actin (BL1039) or ACKR3 (Bioss) at 4°C overnight. Anti‐IgG antibody (CoWin Biotech) was used for detection. Membranes were imaged with Image Lab (Bio‐Rad).

### 2.4 Immunohistochemistry (IHC)

Clinical tumor samples were fixed with 4% paraformaldehyde, embedded in paraffin, and cut into 4 μm sections. Sections were deparaffinized, rehydrated, and the antigens were retrieved with EDTA epitope retrieval buffer (pH 8.0). Horseradish peroxidase (HRP)‐conjugated secondary antibodies (Dako North America Inc.) were used to stain sections after primary antibodies against ACKR3 (1:200, Proteintech) at 4°C overnight. Following the manufacturer's instructions, the DAB Color Development Kit (DAKO) was used to measure antibody expression. The nuclei were counterstained with hematoxylin before the slides were photographed and examined using an optical microscope (Olympus Co.).

### 2.5 Enzyme‐linked immunosorbent assay (ELISA)

The human ACKR3 quantitative ELISA kits (R&D Systems) were used to quantify the serum ACKR3 level following the manufacturer's instructions. Moreover, at 450 nm, the optical density (OD) measurements were recorded.

### 2.6 Cell culture

The human lung adenocarcinoma cell line A549 was acquired from Shanghai Jianglin Biological Technology Co., Ltd. in Shanghai, China, and cultured in RPMI‐1640 medium (GE Healthcare Life Sciences) with 10% heat‐inactivated fetal bovine serum (FBS; Gibco Thermo Fisher Scientific, Inc.), 100 U/mL penicillin, and 0.1 mg/mL streptomycin (Gibco, BRL) at 37°C in a 5% CO_2_ incubator.

### 2.7 Cell transfection

Briefly, A549 cells were seeded in 6‐well plates and transfected when they reached 60% confluency. MiR‐100 mimic, human full‐length ACKR3 cDNA (ovACKR3), their common negative control (Con), or miR‐100 mimic+ovACKR3 (all synthesized by Life Technologies Corporation) were transfected into cells with the Lipofectamine 2000/3000 reagent (Invitrogen). Blasticidin (4 μg/mL)/G418 Sulfate (Geneticin, 250 μg/mL) was applied to select stable miR‐100/ACKR3‐overexpressing clones. The transfection efficiencies were confirmed with qRT‐PCR or WB after 3 weeks of selection.

### 2.8 Animal model (BM of NSCLC)

The Nanchang University Animal Center provided 32 male BALB/c nude mice (4 weeks old) maintained in environments free of pathogens. A549 cells (1 × 10^6^ cells/mouse in 100 μL PBS) transfected with control (Con), miR‐100 mimic, ovACKR3, or miR‐100 mimic+ovACKR3 constructs (*n* = 8 for each group) were used to establish the NSCLC BM+ mice model. Eight weeks after injection, mice brain tissues were harvested for hematoxylin and eosin (H&E) histopathological staining. The incidence of BM was quantified based on the percentage of brain tissue with metastatic NSCLC.

### 2.9 Serum hsa_circ_0072309 treatment

Preoperative serums (5 mL) from BM+ patients with the highest expression of serum hsa_circ_0072309 (1.05), the most advanced TNM stage (IV), and the largest amount of BM (8) were diluted into a series of concentrations (10, 15, 20, 25, and 30%) to make the experimental results more apparent. These were injected via tail vein into NSCLC BM+ mice transfected with Con, miR‐100 mimic, ovACKR3, or miR‐100 mimic+ovACKR3 constructs (*n* = 5 per group). H&E staining was used to quantify the BM in mice brains collected 24 days after injection.

### 2.10 Hematoxylin and eosin (H&E) staining

Mice brain tissues were perfused for 10 min with 10% formalin, harvested, and immersed in 10% formalin overnight. Specimens were embedded in paraffin, sectioned at 5 μm thickness, and stained with the H&E Kit (Sigma‐Aldrich) as described by the manufacturer. Images were observed and photographed under a light microscope (Olympus Co.) for visualization and analysis.

### 2.11 Statistical analysis

Statistical analysis was done using the SPSS software (version 21.0, Inc.). Data are presented as mean ± standard deviation (SD), number (*n*), and rate (%). Student's *t*‐test performed quantitative data comparisons between two groups, while comparisons between multiple groups were performed by one‐way analysis of variance (ANOVA). Categorical data between groups were evaluated with the Chi‐square (*χ* ^2^) test, while ranked data were assessed using the Pearson *χ* ^2^ or non‐parametric tests (Somers' *D*/Kendall's tau‐b). Correlation analysis was performed using Spearman's correlation coefficient analysis, and survival analysis was evaluated with the Log‐rank (Mantel‐Cox) test. *p*‐value < 0.05 was defined as statistically significant.

## 3 RESULTS

### 3.1 Analysis of basic clinical data

As shown in Table 2, the basic clinical data, including age, gender, smoking history, and family history of lung cancer, were not statistically different between NSCLC patients, with and without BM, and healthy volunteers (*p* > 0.05), indicating comparability between groups. Moreover, there was a significant difference between NSCLC BM+ patients (25 III and 35 IV) and NSCLC BM− patients (5 I, 6 II, 27 III, and 22 IV) (*p* < 0.01) in tumor stage (TNM stage), suggesting that patients with NSCLC who develop BM are usually at the advanced stage (III and mostly IV stage).

**TABLE 2:** The basic clinical features were accordant cross‐talk between NSCLC BM+ patients, NSCLC BM− patients, and healthy volunteers.

Parameters	Groups (*n* = 60/group)		*p*‐values	Groups (*n* = 60/group)		*p*‐values	Groups (*n* = 60/group)		*p*‐values
	NSCLC BM+ patients	NSCLC BM− patients		NSCLC BM+ patients	Healthy volunteers		NSCLC BM− patients	Healthy volunteers	
Age (years)									
≤40	34	32	0.714	34	33	0.854	32	33	0.855
>40	26	28		26	27		28	27	
Gender									
Male	42	38	0.439	42	40	0.695	38	40	0.702
Female	18	22		18	20		22	20	
TNM stage									
I	—	5	0.003	—	—	—	5	—	—
II	—	6		—	—		6	—	
III	25	27		25	—		27	—	
IV	35	22		35	—		22	—	
Smoking history									
Yes	36	33	0.580	36	30	0.271	33	30	0.583
No	24	27		24	30		27	30	
Family history of LC									
Yes	24	22	0.672	24	18	0.251	22	18	0.129
No	36	28		36	42		28	42	

Abbreviations: NSCLC, non‐small‐cell lung cancer; TNM, tumor‐node‐metastasis.

### 3.2 Hsa_circ_0072309 and ACKR3 are up‐regulated, and miR‐100 is down‐regulated in BM tissues from NSCLC

**FIGURE 1:**
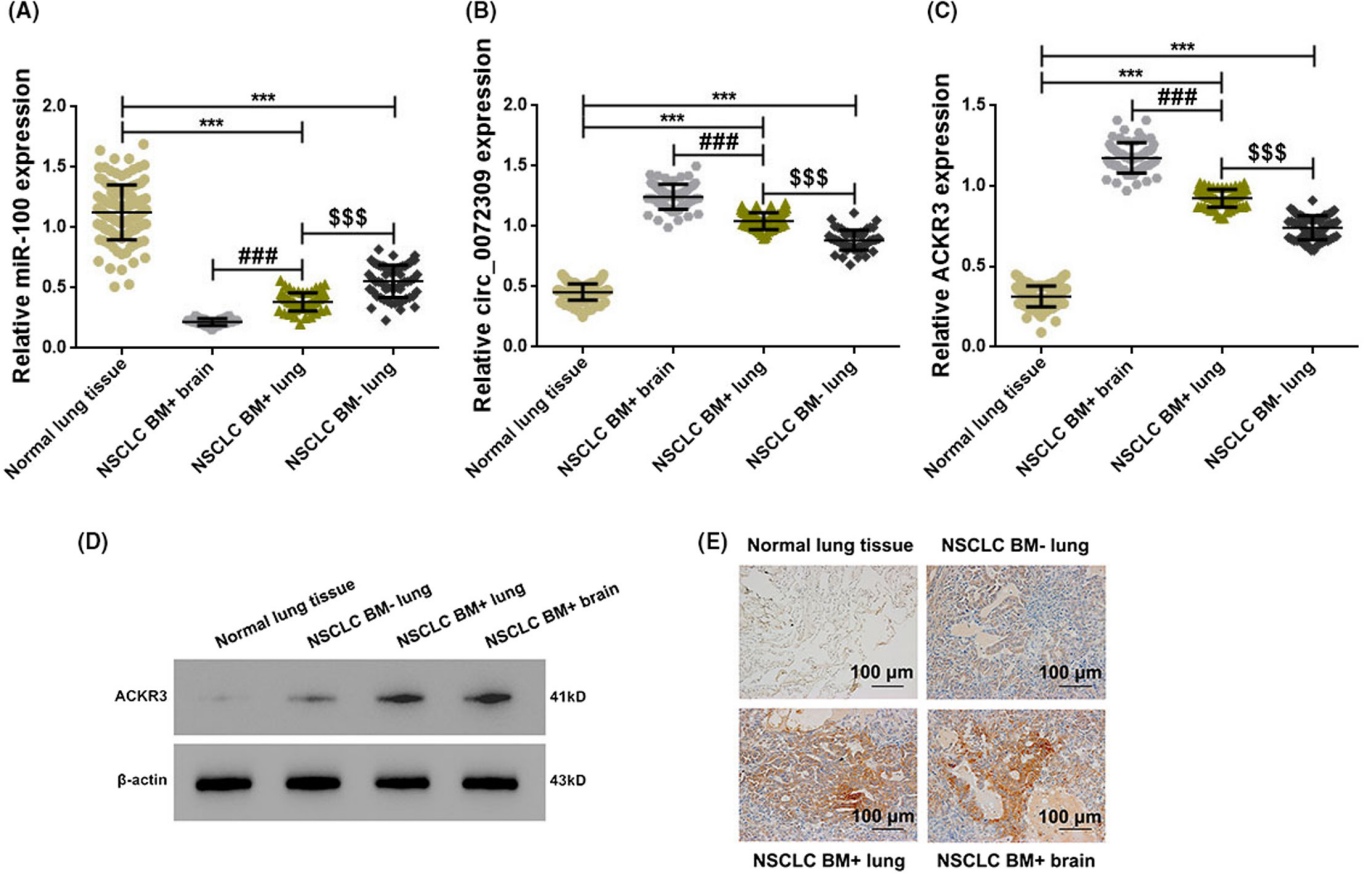
Up‐regulation of hsa_circ_0072309 and ACKR3, and down‐regulation of miR‐100 in brain metastases of NSCLC patients. Graphs showing the relative RNA expression of hsa_circ_0072309 (A), ACKR3 (B), and miR‐100 (C) in clinical tissue specimens from NSCLC patients as detected by qPCR. *** versus Normal lung tissues, *p* < 0.001; ### versus NSCLC BM+ lung tissues, *p* < 0.001; $$$ versus NSCLC BM− lung tissues, *p* < 0.001. (D) Representative Western blot showing the expression of ACKR3 in clinical tissue specimens. (E) Immunohistochemistry staining of ACKR3 in clinical tissue specimens with brown stains indicating positivity (scale bar:100 μm; magnification: ×200).

We first examined the expression of hsa_circ_0072309, ACKR3, and miR‐100 at the gene level by qPCR. Lung tumor samples from both BM+ and BM− patients showed significant up‐regulation in hsa_circ_0072309 and ACKR3, while miR‐100 is downregulated compared to normal peritumor samples (*p* < 0.001; Figure 1A–C). The same trend was observed when comparing lung tumor samples from BM+ and BM− patients, as well as between BM foci and lung tumor foci from BM+ patients (all *p* < 0.001). At the protein level, Western blot analysis showed that both BM+ and BM− patients had higher levels of ACKR3 protein in their lung tumors when compared to the surrounding tissue, in their lung tissues when compared to BM− patients, and in their BM foci when compared to their lung foci (all *p* < 0.001; Figure 1D,E).

### 3.3 Hsa_circ_0072309 and ACKR3 are up‐regulated, and miR‐100 is down‐regulated in preoperative serum specimens of NSCLC BM+ patients

**FIGURE 2:**
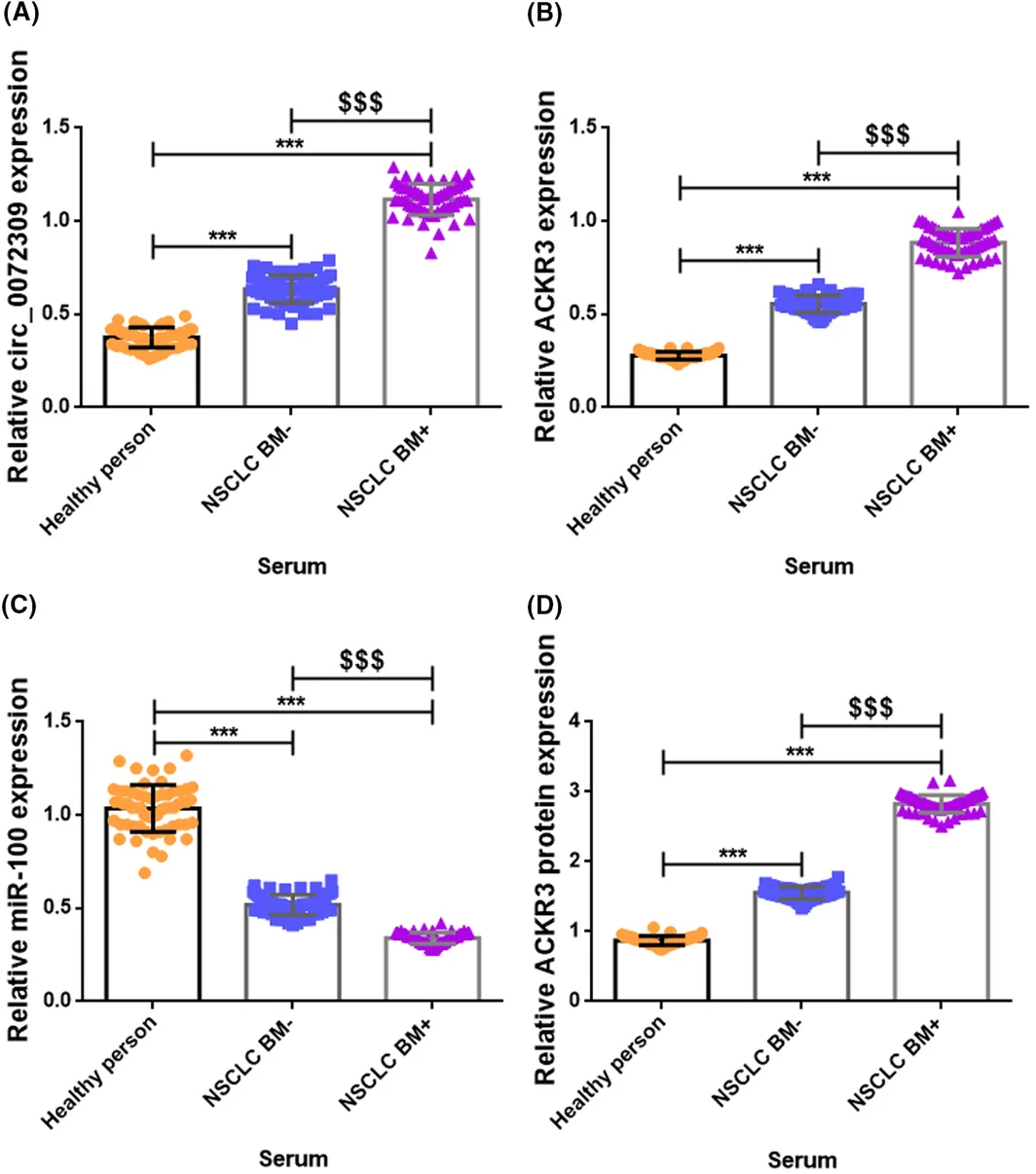
Up‐regulation of hsa_circ_0072309 and ACKR3, and down‐regulation of miR‐100 in preoperative serum specimens from NSCLC BM+ patients. Graphs showing the relative RNA expression of hsa_circ_0072309 (A), ACKR3 (B), and miR‐100 (C) in preoperative serum specimens from BM+ patients as detected by qPCR. (D) Graph showing the expression of ACKR3 protein in serum specimens as detected by ELISA. *** versus Healthy person, *p* < 0.001; $$$ versus NSCLC BM−, *p* < 0.001.

We employed preoperative serum samples from NSCLC patients with and without BM. We compared them to those of healthy controls to do qPCR and ELISA to determine if the differential expression of hsa_circ_0072309, ACKR3, and miR‐100 are expressed in the patient's blood. At the gene level, we found the same trend of increased hsa_circ_0072309 and ACKR3 and reduced miR‐100 in all NSCLC patients compared to healthy volunteers and in BM+ patients compared to BM− patients (all *p* < 0.001; Figure 2A–C). ACKR3 expression in the preoperative serum specimens at the protein level displayed the same trends as its RNA expression results among the comparison groups (*p* < 0.001; Figure 2D).

### 3.4 Differential correlations between hsa_circ_0072309, ACKR3 and miR‐100 in NSCLC patients with BM

**FIGURE 3:**
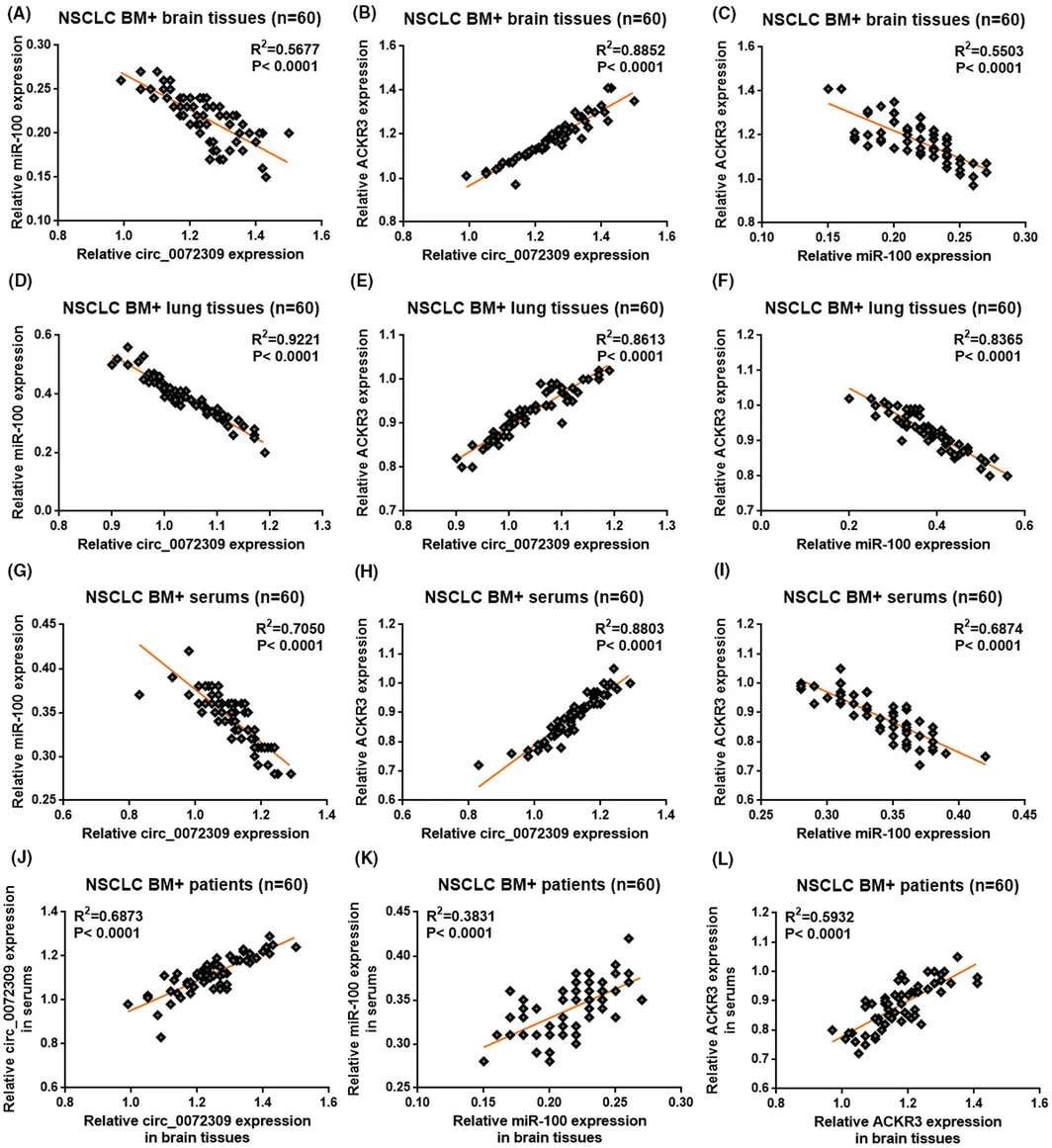
Differential correlations between hsa_circ_0072309, ACKR3, and miR‐100 in NSCLC patients with BM. Graphs showing the Pearson correlation between expressions of hsa_circ_0072309 and ACKR3 (A), hsa_circ_0072309 and miR‐100 (B), miR‐100 and ACKR3 (C) in the brain tissues of BM+ patients. Graphs showing the Pearson correlation between expressions of hsa_circ_0072309 and ACKR3 (D), hsa_circ_0072309 and miR‐100 (E), miR‐100 and ACKR3 (F) in the lung tissues of BM+ patients. Graphs showing the Pearson correlation between expressions of hsa_circ_0072309 and ACKR3 (G), hsa_circ_0072309 and miR‐100 (H), miR‐100 and ACKR3 (I) in the serums of BM+ patients. Graphs showing the Pearson correlation between brain tissue and serum expression of hsa_circ_0072309 (J), ACKR3 (K), miR‐100 (L).

Correlation analyses were conducted to determine how hsa_circ_0072309, ACKR3, and miR‐100 interact. One positive correlation (hsa_circ_0072309 vs. ACKR3) and two negative correlations (hsa_circ_0072309 vs. miR‐100; miR‐100 vs. ACKR3) were observed in the BM samples (Figure 3A–C), primary lung carcinoma foci (Figure 3D–F), and serum samples (Figure 3G–I) of BM+ patients (*p* < 0.0001). Interestingly, there are positive correlations between hsa_circ_0072309/ACKR3/miR‐100 in the BM foci and their counterparts in the serum (*p* < 0.0001; Figure 3J–L).

### 3.5 Serum hsa_circ_0072309 is predictive of disease progression in NSCLC BM+ patients

**FIGURE 4:**
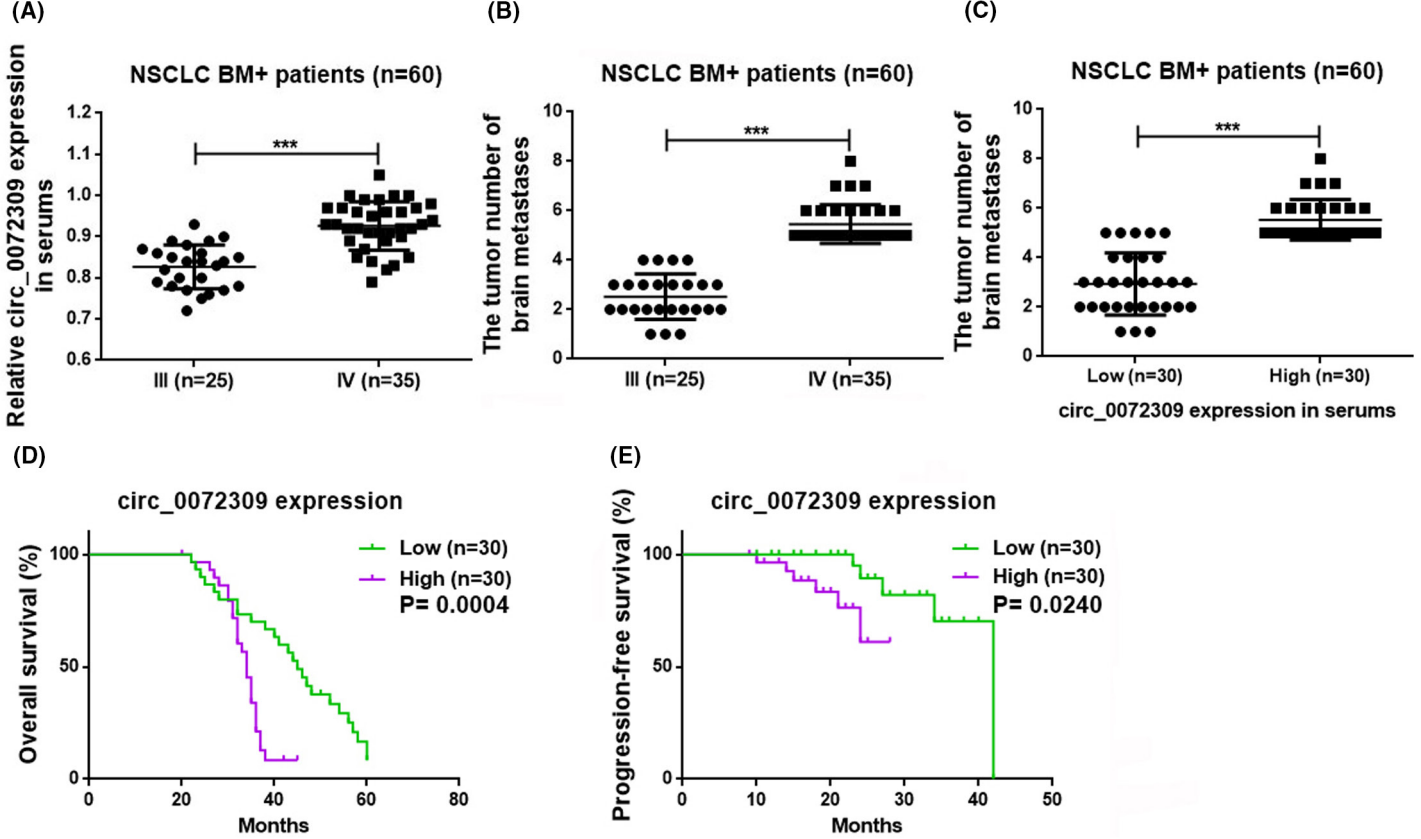
Serum hsa_circ_0072309 predicts disease progression in NSCLC BM+ patients. (A) Graph comparing the serum hsa_circ_0072309 level in BM+ patients with stages III and IV disease. (B) Graph comparing the number of brain metastases in BM+ patients with stages III and IV disease (***p < 0.001). (C) Graph comparing the number of brain metastases in BM+ patients with low and high serum hsa_circ_0072309 levels (***p < 0.001). (D) Graph comparing the overall survival (OS) of BM+ patients with low and high serum circ_0072309 levels. (E) Graph comparing the progression‐free survival (PFS) of BM+ patients with low and high serum circ_0072309 levels.

We investigated the relationship between this circRNA and tumor stage and BM to determine if the expression of hsa_circ_0072309 in the serum predicts the disease state. Serum hsa_circ_0072309 levels are significantly higher in stage IV BM+ patients than in stage III patients (*p* < 0.001; Figure 4A). Since the CT images of stage IV patients show increased BM foci compared to those from stage III patients (*p* < 0.001; Figure 4B), it follows that we also observed more BM foci in patients with high serum hsa_circ_0072309 expression than in those with low expression (low and high expression groups were determined according to the median expression of hsa_circ_0072309 in the serum; *p* < 0.001; Figure 4C). Evaluating the long‐term prognostic value of serum hsa_circ_0072309 level, we found that both the post‐operative OS (*p* < 0.001; Figure 4D) and PFS (*p* < 0.05; Figure 4E) are decreased in patients with high serum hsa_circ_0072309 expression compared to those with low expression at the same post‐operative time points. These findings revealed that serum hsa_circ_0072309 levels are connected to the clinical stage and number of BM foci in NSCLC BM+ patients, with later clinical stages being associated with more BM foci and higher serum hsa_circ_0072309 levels. Because of the association with a worse prognosis, hsa_circ_0072309 levels may be a biomarker for NSCLC advancement.

### 3.6 ALK mutation did not affect the prognosis of NSCLC patients with BM, and the expressions of hsa_circ_0072309, miR‐100, and ACKR3 mRNAs and ACKR3 protein in serum

**FIGURE 5:**
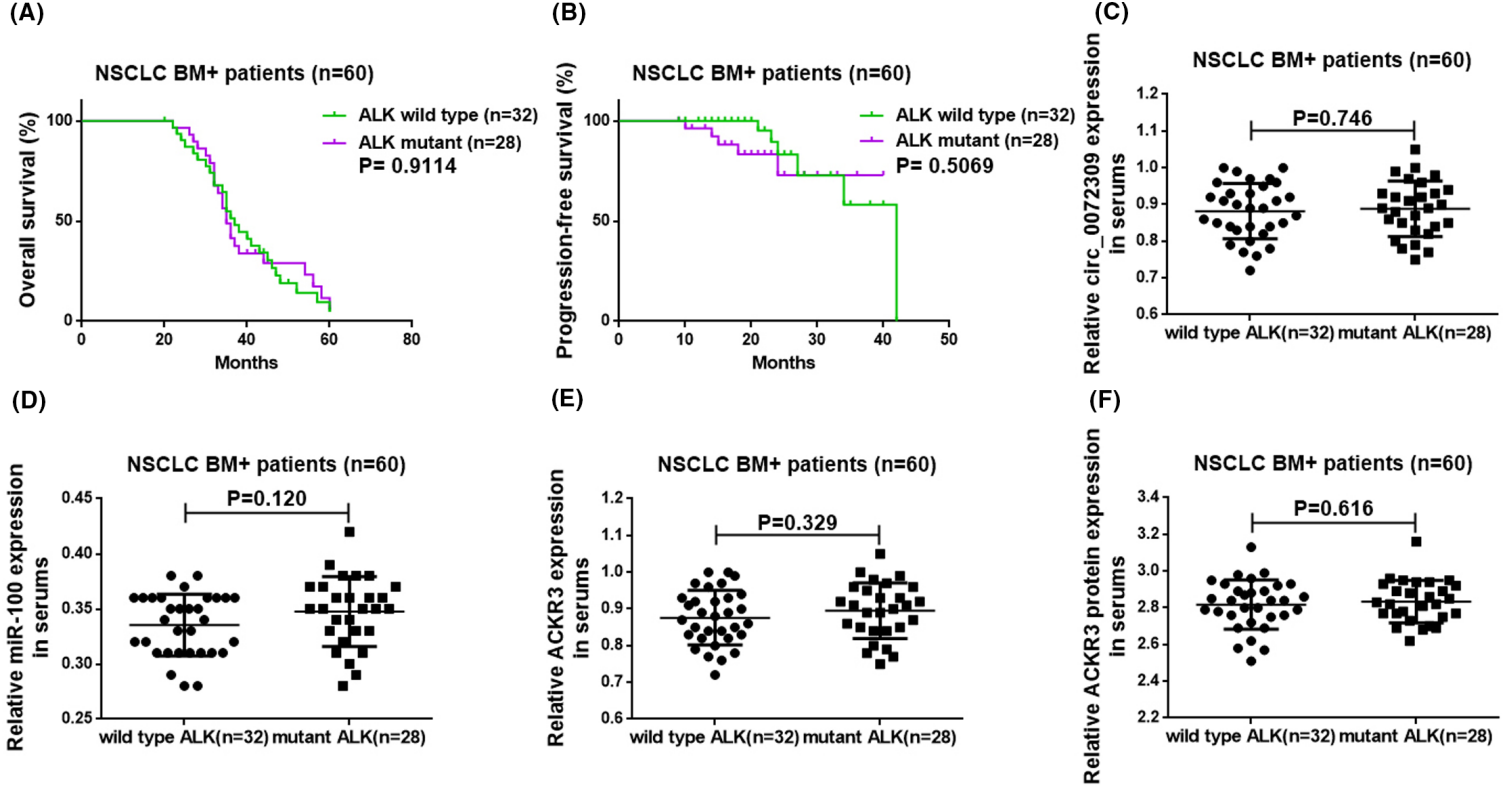
ALK mutation did not affect the prognosis of NSCLC BM+ patients and the expressions of hsa_circ_0072309, miR‐100, and ACKR3 mRNAs and ACKR3 protein in serum. (A, B) The roles of ALK mutation on post‐operative OS and PFS of NSCLC BM+ patients, respectively. (C–F) The influences of ALK mutation on serum hsa_circ_0072309, miR‐100, and ACKR3 mRNA levels, and ACKR3 protein level in NSCLC BM+ patients.

We compared genetic mutation information between NSCLC patients with and without BM to rule out the influence of genetic mutations on the outcomes. NSCLC patients with BM showed 60 harboring wild‐type EGFR and mutant KRAS, 32 wild‐type ALK and 28 mutant ALK. In comparison, NSCLC patients without BM showed 60 harboring wild‐type EGFR and mutant KRAS, 30 wild‐type ALK and 30 mutant ALK, ensuring similarity between patients from the two groups. Since we mainly considered the role and possible mechanism of serum hsa_circ_0072309 in BM arising from NSCLC, we primarily analyzed whether ALK gene mutation affected the prognosis of NSCLC BM+ patients and whether it was related to serum hsa_circ_0072309/miR‐100/ACKR3 axis. The results showed that in NSCLC BM+ patients with wild‐type and mutant ALK, the post‐operative OS and PFS, serum levels of hsa_circ_0072309, miR‐100, and ACKR3 mRNA and protein were not statistically different (*p* > 0.05; Figure 5), indicating that ALK mutation had no impact on our experimental findings.

### 3.7 Hsa_circ_0072309‐stimulated BM can be reduced by regulating the miR‐100/ACKR3 signaling axis

**FIGURE 6:**
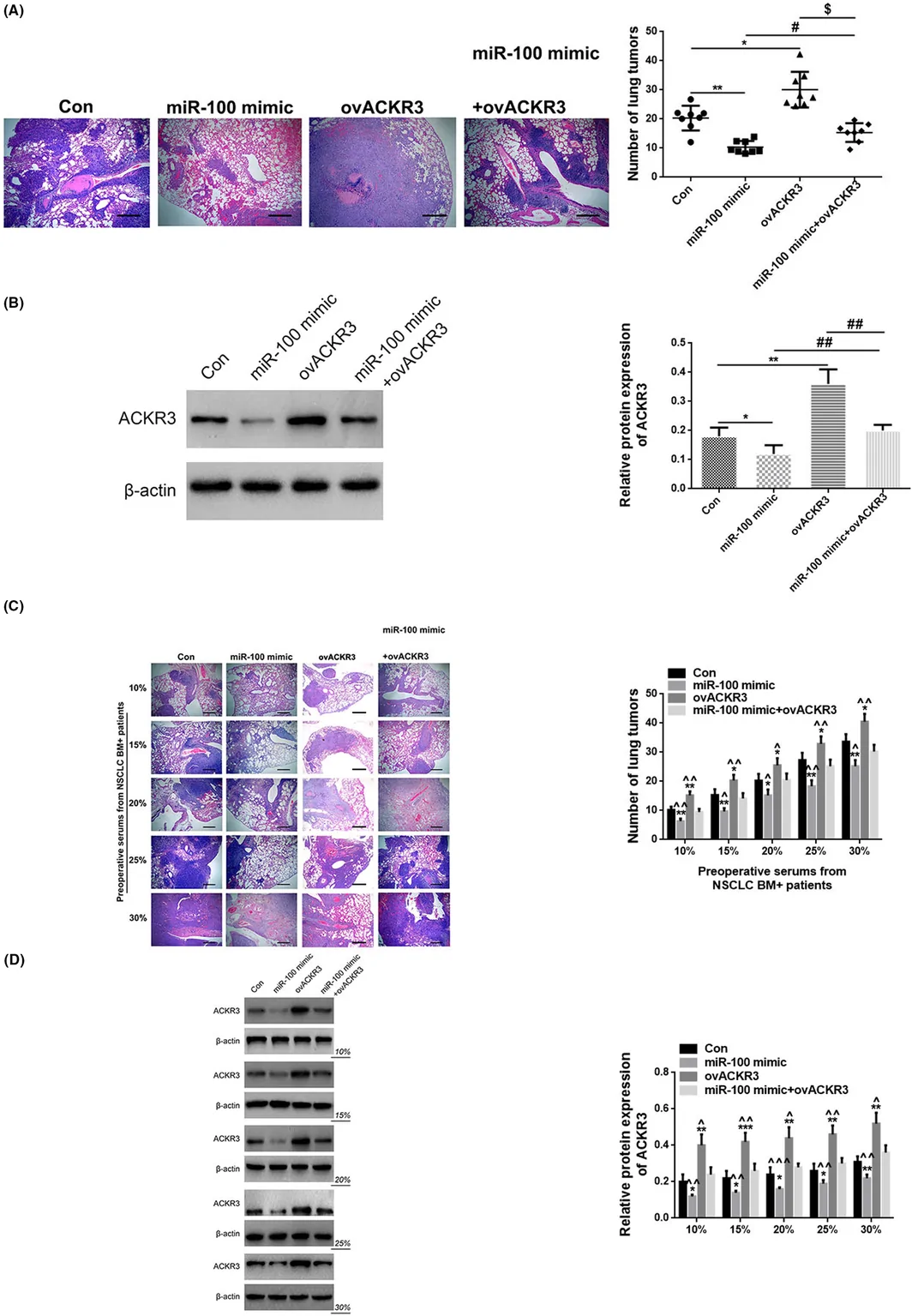
Hsa_circ_0072309‐stimulated BM can be reduced by regulating the miR‐100/ACKR3 signaling axis in vivo. (A) Representative H&E staining of metastatic lung tumors in the brain (left) and quantification of these tumors (right) in mice transplanted with A549 cells expressing control cDNA (Con), miR‐100 mimic, ACKR3 cDNA (ovACKR3) or miR‐100 mimic + ovACKR3. magnification: ×200. * versus con, *p* < 0.05; ** versus con, *p* < 0.01; # versus miR‐100 mimic, *p* < 0.05; $ versus ovACKR3, *p* < 0.05. (B) Relative protein expression of ACKR3 in mice at Con, miR‐100 mimic, ovACKR3, or miR‐100 mimic + ovACKR3 group. * versus con, *p* < 0.05; ** versus con, *p* < 0.01; ## versus miR‐100 mimic + ovACKR3, *p* < 0.01. (C) Graph showing the number of BM in mice (Con, miR‐100 mimic, ovACKR3, and miR‐100 mimic + ovACKR3) treated with increasing doses of hsa_circ_0072309‐containing preoperative serum from NSCLC BM+ patients. magnification: ×200. * versus con, *p* < 0.05; ** versus con, *p* < 0.01; ^ miR‐100 mimic + ovACKR3, *p* < 0.05; ^^ miR‐100 mimic + ovACKR3, *p* < 0.01. (D) The protein expression of ACKR3 in mice (Con, miR‐100 mimic, ovACKR3, and miR‐100 mimic + ovACKR3) treated with increasing doses of hsa_circ_0072309‐containing preoperative serum from NSCLC BM+ patients. * versus con, *p* < 0.05; ** versus con, *p* < 0.01; *** versus con, *p* < 0.01; ^ miR‐100 mimic + ovACKR3, *p* < 0.05; ^^ miR‐100 mimic + ovACKR3, *p* < 0.01; ^^^ versus miR‐100 mimic + ovACKR3, *p* < 0.001.

We developed a BM model using the A549 lung tumor cell line to examine whether hsa_circ_0072309, ACKR3, and miR‐100 can interact in vivo to regulate the development of NSCLC‐derived BM. Mice transplanted with A549 cells expressing a miR‐100 mimic formed fewer BM than control mice (*p* < 0.01), but animals transplanted with ACKR3 overexpressing cells (ovACKR3) developed more BM, according to H&E staining of brain tissues (*p* < 0.05). Moreover, compared with the miR‐100 mimic+ovACKR3 group, the number of BM in mice with tumor cells expressing a miR‐100 mimic was decreased (*p* < 0.05), while the expression of ovACKR3 was increased (*p* < 0.05). Still, the number of BM in mice with tumor cells expressing a miR‐100 mimic+ovACKR3 was similar to the control (*p* > 0.05), indicating that miR‐100 expression can negate the effect of ACKR3 overexpression (Figure 6A). To evaluate the effect of hsa_circ_0072309, we showed that preoperative serum from NSCLC BM+ patients with a high concentration of this circRNA could increase the number of BM in mice in the same group in a concentration‐dependent manner (Figure 6C). Mice transplanted with cells co‐expressing miR‐100 mimic and ovACKR3 were able to partially reverse the downward trend in BM (15%, 20%, and 30% serum, *p* < 0.05; the other concentration of serum, *p* < 0.01) when compared to the Con group (20% serum, *p* < 0.05; the other concentration of serum, *p* < 0.01) under the same serum infusion concentrations (Figure 6C). The mice transplanted with ovACKR3 cells showed increased BM compared with the Con group (10% serum, *p* < 0.01; the other concentration of serum, *p* < 0.05), and mice transplanted with cells co‐expressing miR‐100 mimic and ovACKR3 could partially reverse the upward trend (20% serum, *p* < 0.05; the other concentration of serum, *p* < 0.01; Figure 6C). WB of brain tissues showed that the protein expression of ACKR3 was inhibited by overexpression of miR‐100 (*p* < 0.05) and promoted by overexpression of ACKR3 (*p* < 0.01). Comparing the suppressed or promoted effect to the co‐overexpression of miR‐100 and ACKR3, the effect could be decreased (*p* < 0.01) (Figure 6B). Homoplastically, preoperative serum from NSCLC BM+ patients with a high concentration of hsa_circ_0072309 can also increase the ACKR3 protein level in mice in the same group in a concentration‐dependent manner (Figure 6D). Under the same serum infusion concentrations, mice transplanted with miR‐100 mimic cells showed declined ACKR3 protein expression compared with the Con group (30% serum, *p* < 0.01; the other concentration of serum, *p* < 0.05), and mice transplanted with cells co‐expressing miR‐100 mimic and ovACKR3 could markedly reverse the downward trend (20% serum, *p* < 0.001; the other concentration of serum, *p* < 0.01; Figure 6D). The mice transplanted with ovACKR3 cells exhibited enhancive ACKR3 protein expression compared with the Con group (15% serum, *p* < 0.001; the other concentration of serum, *p* < 0.01), and mice transplanted with cells co‐expressing miR‐100 mimic and ovACKR3 could partially reverse the upward trend (15% and 25% serum, *p* < 0.01; the other concentration of serum, *p* < 0.05; Figure 6D).

## 4 DISCUSSION

Despite advancements in the development of numerous therapeutic options, BM—the most frequent incidence in LC patients—is a significant factor in treatment failure, a poor prognosis, and mortality.^7^, ^25^ The clinical symptoms of BM arising from NSCLC usually appear late, and there is considerable heterogeneity among patients.^26^ Currently, imaging methods like CT and MRI are largely used to diagnose BM. However, these methods frequently struggle to correctly identify small BM lesions, and only 10%–18% of patients receive a CT diagnosis, while 24% of patients receive an MRI diagnosis.^27^ Moreover, diagnostic imaging costs are high, and patient compliance is low. As a result, it is difficult to observe a patient's state dynamically using various modalities for continuous monitoring. Therefore, developing simple, accurate, rapid, and economical detection strategies for early diagnosis, therapeutic monitoring, and prognostic evaluation of LC‐associated BM is crucial. In recent years, with the development of molecular biology, many new biomarkers have been discovered and studied in tumor tissues, peripheral blood, cerebrospinal fluid, and other clinical specimens of LC patients with BM.^6^, ^28^, ^29^ In the present study, we established that hsa_circ_0072309 is up‐regulated in the tissue and serum of LC patients with BM and that serum hsa_circ_0072309 level is strongly correlated with clinical stage, BM foci and poor prognosis in NSCLC BM+ patients. In addition, serum hsa_circ_0072309 can promote BM by regulating the miR‐100/ACKR3 pathway in NSCLC mice. These findings indicate that serum hsa_circ_0072309 may be a feasible biomarker for diagnosing, prognosis, and treating NSCLC‐related BM.

LC is more frequently observed in males and smokers.^30^ Unfortunately, most LC patients have clinically advanced stages when first identified, and many usually present with BM.^31^ Moreover, a higher proportion of patients with LC/NSCLC‐derived BM are young (≤40 years) and harbors driver gene mutations (family history of LC) in their tumors.^30^, ^32^ So, at the beginning of this research, we specifically analyzed the basic clinical data, including age, sex, tumor stage, smoking history, and family history of our patients. Except for the TNM stage, no significant differences were observed between groups, indicating patient comparability.

Circular RNAs (circRNAs) are a recently discovered novel subclass of endogenous non‐coding RNAs.^33^ Unlike linear RNAs, circRNAs form a covalent continuous closed loop structure,^34^ stabilizing them and increasing their resistance to RNase degradation.^33^ Most circRNAs possess specific patterns of expression depending on cell and tissue type and developmental stage.^35^, ^36^ CircRNAs are believed to function by sponging miRNA, which inhibits miRNA expression and enhances the expression of genes targeted by miRNA in various biological processes and diseases, including cancer.^37^, ^38^ Importantly, an increasing number of researchers have uncovered that circRNAs may potentially be promising diagnostic biomarkers and therapeutic targets for multiple diseases, including NSCLC,^13^ where overexpressed of some circRNAs including hsa_circRNA_100876,^39^ hsa_circ_0013958,^40^ and hsa_ circ_0014130^41^ have been validated.

We verified that hsa_circ_0072309 and ACKR3 are up‐regulated in the current study. MiR‐100 expression is also decreased in NSCLC BM+ patients' blood, BM tissues, and original lung tumor specimens compared to BM− patients, healthy volunteers, or normal surrounding peritumor tissues. Further, the same trends can be found when comparing BM tissues to primary LC tissues in NSCLC BM+ patients. These results suggest that overexpression of hsa_circ_0072309/ACKR3 or downregulation of miR‐100 may promote metastasis of primary lung tumors to the brain in NSCLC patients. Earlier studies have revealed that hsa_circ_0072309 is significantly overexpressed in human NSCLC tissues when compared to matching normal samples and elevated in A549 LC cells when compared to BEAS‐2B normal lung cells, where it promotes NSCLC cell proliferation, migration, and invasion while inhibits apoptosis.^42^, ^43^ Similarly, CXCR7 (ACKR3) is overexpressed in lung tumor tissues, and upregulation of CXCR7 markedly promotes A549 cell migration in vitro and enhances tumor growth and metastasis in vivo.^44^, ^45^ In contrast, miR‐100 is substantially downregulated in NSCLC tissues, and miR‐100 restoration can inhibit the formation of tumors, the advancement of the G2/M cell cycle, and the proliferation, invasion, and migration of NSCLC cells.^20^, ^46^, ^47^ Low miR‐100 expression is correlated to old age, higher clinical stage, advanced tumor classification, lymph node metastasis, and lower OS in NSCLC patients, indicating a possible prognostic role for this microRNA in NSCLC.^20^, ^47^ These observations are consistent with our most recent findings regarding the reciprocal expression of miR‐100, ACKR3, and hsa_circ_0072309.

Additionally, our study team observed that the expression of hsa_circ_0072309 is favorably correlated with ACKR3 and negatively correlated with miR‐100. At the same time, in the BM, primary LC tissues, and serum of NSCLC BM+ patients, miR‐100 level is adversely linked with ACKR3. Further, BM expression of hsa_circ_0072309/miR‐100/ACKR3 positively correlates with their counterparts in the serum for NSCLC BM+ patients. Previous studies show that CXCR7 (ACKR3) is a direct gene target of miR‐100 in esophageal squamous cancer cells^19^ and miR‐100 is a direct miRNA target of hsa_circ_0072309 in ischemic stroke patients.^23^ Our animal experiments corroborate the conclusion that ACKR3 is regulated by miR‐100, which is regulated by hsa_circ_0072309, even though we did not further validate the relationship between these RNAs/genes. We discovered that overexpressing miR‐100 repressed while overexpressing ACKR3 accelerated the production of BM from NSCLC cells, and co‐transfection of both molecules neutralized their individual effects, indicating that miR‐100 limits BM development by regulating ACKR3. Preoperative serum from BM+ patients with high hsa_circ_0072309 expression can promote BM formation in a concentration‐dependent manner. This promotive effect was relieved by expressing the miR‐100 mimic, while upregulation of ACKR3 partially reversed the repressive role of the miR‐100 mimic. These findings indicate that serum hsa_circ_0072309 overexpression facilitates NSCLC‐related BM by modulating the miR‐100/ACKR3 axis.

Finally, we demonstrated a strong correlation between high serum hsa_circ_0072309 concentration, advanced TNM stage, more BM foci, and poorer post‐operative OS and PFS. According to a prior study, ACKR3 overexpression can serve as a biomarker for NSCLC patients' poor post‐operative 5‐year PFS and recurrence.^15^ Low miR‐100 expression also predicts higher clinical stage, advanced tumor classification, lymph node metastasis, and lower OS in NSCLC patients.^20^, ^47^ However, no studies have been published on using serum hsa_circ_0072309 levels in the diagnosis, prognosis, or treatment of NSCLC‐derived BM. Our findings indicate that circulating serum hsa_circ_0072309, which acts via miR‐100/ACKR3 signaling, could be a new diagnostic and prognostic biomarker and a possible therapeutic target for LC metastatic transformation.

## 5 CONCLUSIONS

In summary, the miR‐100/ACKR3 pathway is regulated by hsa_circ_0072309, up‐regulated in primary lung tumors, brain metastases, and serum of NSCLC patients with BM. Serum hsa_circ_0072309 may be a potential diagnostic and prognostic biomarker and a therapeutic target for BM in NSCLC patients, which requires further investigation.

## AUTHOR CONTRIBUTIONS

**Xiao‐Qiang Zhang:** Data curation (lead); writing – original draft (equal). **Qian Song:** Methodology (lead); validation (equal). **Lin‐Xiang Zeng:** Project administration (lead); writing – review and editing (lead).

## FUNDING INFORMATION

There is no specific funding to support this research.

## CONFLICT OF INTEREST STATEMENT

There are no potential conflicts of interest among the authors.

## ETHICS APPROVAL AND CONSENT TO PARTICIPATE

The clinical study protocol was authorized by the Ethical Commission of the Second Affiliated Hospital of Nanchang University, and the research procedures were conducted following the provisions of The Code of Ethics of the World Medical Association (Declaration of Helsinki, 2008) and the International Ethical Guidelines for Biomedical Research Involving Human Subjects (2002). Written informed consent was obtained from all participants before the start of this study. In addition, all animal experiments complied with the Second Affiliated Hospital of the Nanchang University of Medicine Policy on Care and Use of Laboratory Animals, the ARRIVE guidelines, and were carried out under the UK Animals (Scientific Procedures) Act, 1986 and associated procedures, EU Directive 2010/63/EU for animal experiments, or the National Research Council's Guide for the Care and Use of Laboratory Animals.

## Data Availability

All data produced or analyzed in the current research are included within this paper.
